# Gaps in artificial intelligence research for rural health in the United States: a scoping review

**DOI:** 10.1093/jamia/ocaf206

**Published:** 2025-11-24

**Authors:** Katherine E Brown, Sharon E Davis

**Affiliations:** Department of Biomedical Informatics, Vanderbilt University Medical Center, Nashville, TN 37203, United States; Department of Biomedical Informatics, Vanderbilt University Medical Center, Nashville, TN 37203, United States

**Keywords:** artificial intelligence, machine learning, large language models, rural healthcare, US healthcare

## Abstract

**Objective:**

Artificial intelligence (AI) has impacted healthcare at urban and academic medical centers in the US. There are concerns, however, that the promise of AI may not be realized in rural communities. This scoping review aims to determine the extent of AI research in the rural US.

**Materials and Methods:**

We conducted a scoping review following the PRISMA guidelines. We included peer-reviewed, original research studies indexed in PubMed, Embase, and WebOfScience after January 1, 2010 and through April 29, 2025. Studies were required to discuss the development, implementation, or evaluation of AI tools in rural US healthcare, including frameworks that help facilitate AI development (eg, data warehouses).

**Results:**

Our search strategy found 26 studies meeting inclusion criteria after full text screening with 14 papers discussing predictive AI models and 12 papers discussing data or research infrastructure. AI models most often targeted resource allocation and distribution. Few studies explored model deployment and impact. Half noted the lack of data and analytic resources as a limitation. None of the studies discussed examples of generative AI being trained, evaluated, or deployed in a rural setting.

**Discussion:**

Practical limitations may be influencing and limiting the types of AI models evaluated in the rural US. Validation of tools in the rural US was underwhelming.

**Conclusion:**

With few studies moving beyond AI model design and development stages, there are clear gaps in our understanding of how to reliably validate, deploy, and sustain AI models in rural settings to advance health in all communities.

## Introduction

A commonly understudied health inequality in biomedical informatics is the divide in access to informatics advances between urban and rural areas in the United States. The dissemination of informatics tools such as healthcare artificial intelligence (AI) to rural communities has critical implications. For example, commonly used sepsis detection models^1^ and hospital readmission models^2^ have demonstrated variation in discrimination and calibration performance across facilities within the same healthcare system. Such differences in performance may translate to differential accrual of the benefits of AI-enabled care across communities receiving care in urban and rural areas. Rural communities, while sparsely populated compared to cities, represent ∼56 million people (∼18% of the US population) based on Centers for Medicare and Medicaid Services (CMS) designation of rural and borderline rural areas.^3^ While the existence of multiple classification measures defining rurality complicates studies of rural areas, the simplest definition in the US is an area (eg, ZIP code tabulation area, county) that is not considered urban.^4^ For example, rurality can be defined at the county-level through the use of Rural-Urban Continuum Codes (RUCC),^5^ which range between 1 indicating highly urban areas and 9 indicating highly rural areas. Using the common classification scheme of rural counties having RUCC scores of 4-9,^5^^,^^6^ ∼43 million people live in rural communities and 61% of US counties are deemed rural.^6^ All US states except New Jersey and Rhode Island contain at least one rural county and in 36 states at least half of the counties are designated as rural. Thus any delay in or barrier to the diffusion of healthcare AI innovation to rural communities leaves a considerable gap and millions of patients that may not benefit from emerging technologies.

Rural populations in the US face a growing barrage of differences in care and health outcomes compared to their urban counterparts. This includes, for example, higher mortality rates for trauma,^7^ stroke care,^8^ and cancer^9^ than urban areas. There are multiple potential factors that may contribute to this disparity. First, there is a shortage of general practice^10–12^ and specialty medicine^13–15^ physicians working in rural areas. This limits the ability of rural populations to access necessary medical care in a timely fashion and for the limited number of providers to effectively serve patients. Hospitals and medical centers in rural America have also been closing at alarming rates.^16^ These hospital closures not only further impact ease and availability of healthcare access^17^^,^^18^ but also have a negative economic impact^19^ on the surrounding community. This could further limit local residents’ ability to access care and potentially perpetuate negative health outcomes. Under recent legislation, Medicaid payments—which have been vital to sustained operation of rural and community medical centers—are scheduled to be massively reduced, which researchers estimate will result more medical center closures for these already vulnerable communities.^20^

Given the health disparities and limited healthcare resource in rural areas, advances in care delivery, diffusion of knowledge, and increased efficiencies enabled by innovative AI tools may be particularly beneficial and essential to rural healthcare providers and communities. Yet, in addition to the rural-urban healthcare divide, there is growing evidence across the country and world that there is a *rural-urban digital divide*^21^ with respect to adoption of nascent technology such as broadband internet^22–24^ and electronic health records (EHR).^25^ Moreover, rural communities have historically been associated with late majority and laggards in adopting technological innovations by theories such as the diffusion of innovation.^26^ Thus, we seek to better understand whether this rural-urban digital divide is impacting the development and adoption of AI tools in rural healthcare facilities.

Artificial intelligence—herein defined as discriminative models such as machine learning (ML) *and* generative models such as large language models (LLMs)—promises to enhance clinical care. Our focus is on clinical interactions with AI tools to support patient care and enable clinical practice. Some examples, among many, include improved diagnostics^27^^,^^28^ increased care efficiency,^29^ and reductions in provider workloads.^30^ While these benefits are particularly needed in rural communities, AI development and deployment of have thus far primarily impacted healthcare at urban and academic medical centers. Current research in improving reliability and applicability of AI models in clinical settings is also evolving primarily through work in urban and academic medical centers. Recent research from Stanford University^31^ explored the end-to-end development of an automated system to deploy AI models based on clinician request in the EHR. Stanford University^31^ and Vanderbilt University Medical Center,^32^ among others, are already focusing on sustainable AI solutions by developing and evaluating systems that monitor deployed AI in real-time to identify concerns such as performance and fairness drift. While these developments and research may provide insights and tools that could be leveraged in rural care, the specific needs for smaller and community medical centers may be overlooked in work aimed at deployments in urban and academic facilities and this oversight result in additional disparities in the ability of rural patients to access and receive the highest possible quality of care.

We posit that simply applying existing AI models from urban or academic medical centers alone may not be enough to provide rural areas with sustainable, equitable access to healthcare AI. First, AI models are known to not perform as well at when transferred to new sites with distinct patient populations and care practices.^33^ Moreover, performance of AI models can change over time.^34^^,^^35^ Given the relative sparsity of patients at any one rural site and the likelihood that these sites may not have the appropriate analytic resources, rural medical centers may not have capacity to localize models developed elsewhere, to train their own models, design locally responsive implementation strategies, or maintain models over time.^36^ Additionally, the distinct patient populations in rural areas may have different distributions of demographic, environmental, and clinical variables.^37^ Models developed on urban populations may also underrepresent rural-centric subgroups and fail to include critical exposures unique to rural communities. Such underrepresentation and misspecification may cause these models to have lower overall performance when transferred to rural sites, known as hidden stratification.^38^^,^^39^ Given the fundamental differences between urban and rural communities, there is a substantial need for AI models, methods, and best practices to be specifically developed and optimized for rural areas.

The current focus on AI deployments in urban areas and the history of US urban-rural digital divides raises concerns that the promise of AI may not be realized in rural communities. We focus on the US due to the combination of advancements in biomedical AI and clinical informatics that has occurred alongside limited, national adoption of health information exchanges despite an environment of decentralized and independent healthcare institutions. Thus, a more thorough understanding of the current state and barriers to use of AI in rural care facilities is essential for the medical and public health communities to advance the health of rural populations and reduce geographic health disparities. We conducted a scoping literature review to answer the following research questions: How has research in AI for rural healthcare evolved? For what tasks and with which techniques has AI been developed or evaluated in the rural US? What gaps, if any, exist with the application of AI in rural US healthcare? What challenges, if any, limit the development, implementation, or evaluation of AI in the rural US?

## Materials and Methods

The PRISMA scoping review checklist (PRISMA-ScR)^40^ can be found in Table S1. An Open Science Foundation repository is available at the following link: https://osf.io/rz2xe/? view_only=66f708fb7c6b4cfda582c7d1e2e5378d

### Search strategy and eligibility criteria

We searched PubMed, Embase, and WebOfScience for literature describing AI development or use at medical centers in the rural United States. We broadly define medical center as any organization providing inpatient or outpatient medical care. Table S2 provides the full queries used to retrieve literature for review using key terms such as “rural health” and “artificial intelligence,” “data science,” or “clinical decision support.” We included peer-reviewed, original research studies indexed after January 1, 2010 and through our search date of April 29, 2025. We opted to use January 1, 2010, as the start date of our search to align with passage of the HITECH Act legislating adoption of EHR systems.^41^ Studies were required to discuss the development, implementation, or evaluation of AI tools in rural US healthcare, including frameworks that facilitate AI development (eg, data warehouses). We consider AI technologies that are developed in the rural US or developed outside the rural US and applied or validated in the rural US. We included both EHR-based implementations and non-EHR-based implementations. To ensure papers were not excluded due to using a non-standard definition of rurality, we included papers based on self-declared development or validation in a rural area in a state or territory of the United States. This strategy also accounts for organizational constraints possibly preventing detailed disclosure of site locations that are indeed rural. Table S3 provides the population-concept-context table for this review.

After a pre-screening review of selected titles, we discovered 2 papers that would likely be cited by titles relevant to this scoping review. The first was an editorial by Cecchetti,^42^ and the second a research article detailing the development of a clinical data resource that explicitly includes the rural US.^43^ Thus, any studies citing either were also included for screening.

### Data charting and synthesis

We used Covidence^44^ to facilitate the organization, title and abstract screening, and full-text screening of references. Covidence tracks all references throughout the review process, enables adjudication of discrepancies among reviewers, and summaries the screening process for reporting of results. After removing duplicates, each abstract was screened for eligibility by both authors and discrepancies discussed for consensus. For studies with eligible abstracts, the full text was screened for eligibility by both authors, with any reasons for exclusion noted and discrepancies again discussed until we reached consensus.

### Data extraction and analysis

For those studies deemed eligible after full text screening, we extracted publication, geographic, clinical, and AI model information. We identified these data points to (1) quantify the maturity of AI in the rural US, (2) elucidate a clearer understanding of data resources used or available for use, and (3) understand how rurality is captured in the AI literature. We collected publication year and type (eg, conference proceedings, journal article). We documented the state (if available) and geographic region (derived from state as necessary) of the first author’s institution, last author’s institution, origin of data, and location at which the AI tool was developed, evaluated, or implemented. For external models evaluated as a baseline or comparison at a rural medical facility, we did not collect information on the original model development location. We also collected information regarding the medical specialty and clinical task for which the AI was developed or applied. Additionally, we extracted information regarding what, if any, formal definition of rurality was used in each study. If this information was not available, we determined if enough information was disclosed such that a determination of rurality based on RUCC codes, CMS ambulance fee schedule, or population density could be estimated (see Table 1). Non-rurality determinations by our team did not disqualify studies from inclusion if the study authors stated the work was conducted in a rural area.

**Table 1.** Summarization of definitions of rurality.

Name	Possible values/interpretation	Definition
RUCC	Categorical. 1-9. 1—most urban 9—most rural	Categorization of US counties by urbanization.
RUCA codes	Categorical. 1-9. 1—most urban 9—most rural	Categorization of sub-county census tracts in US by proximity of urban areas.
Population density	Numerical. Lower values are associated with areas more likely to be rural.	Measured as the ratio of people per unit of land area (ie, square miles).
HRSA eligibility	Categorical. Yes/No Rurality of geographical location is one factor for eligibility.	Denotes eligibility of location for a rural health grant from the Health Resources and Services Administration.
Urban influence code	Categorical. 1-9. 1—most urban 9—most rural	Categorization of US counties by urbanization.

In each study, we determined if the AI developed or evaluated was predictive (eg, a ML model predicting a diagnosis or clinical prognosis) or generative (eg, a LLM generating clinical summaries or extracting information from clinical notes) along with the underlying learning algorithm (eg, random forest, gradient boosted tree, neural network, GPT-3.5). We collected details on the evaluation strategy for the AI model and whether the work was an implementation study. We also determined which stage(s) of the AI lifecycle defined by De Silva and Alahakoon^45^ were reported in the study. We consolidated the 19 detailed stages of the proposed AI lifecycle^45^ into 3 broad categories: design (ie, problem formulation and data acquisition), develop (ie, model develop and initial evaluation), and deploy (ie, model deployment, evaluation, and monitoring). We noted the type of data used in the AI (eg, structured EHR data, clinical text) and determined if the data was from a single rural medical center, multiple medical centers (eg, across multiple institutions affiliated with an academic medical center), or a nationwide cohort (eg, All of Us^46^). Finally, we collected any information related to limitations or barriers to AI in rural healthcare as disclosed in the “Discussion” or “Limitations” sections of each paper.

We used Google Forms to facilitate consistent extraction of relevant details and exported all data for analysis in Python. All plots were generated with Matplotlib and GeoPandas.

## Results

First, we present general findings across all papers retrieved in our scoping review. We then consider results from papers considering AI model research and infrastructure research separately. Finally, we consider challenges noted across all papers retrieved from our study.

### General findings

**Figure 1.**
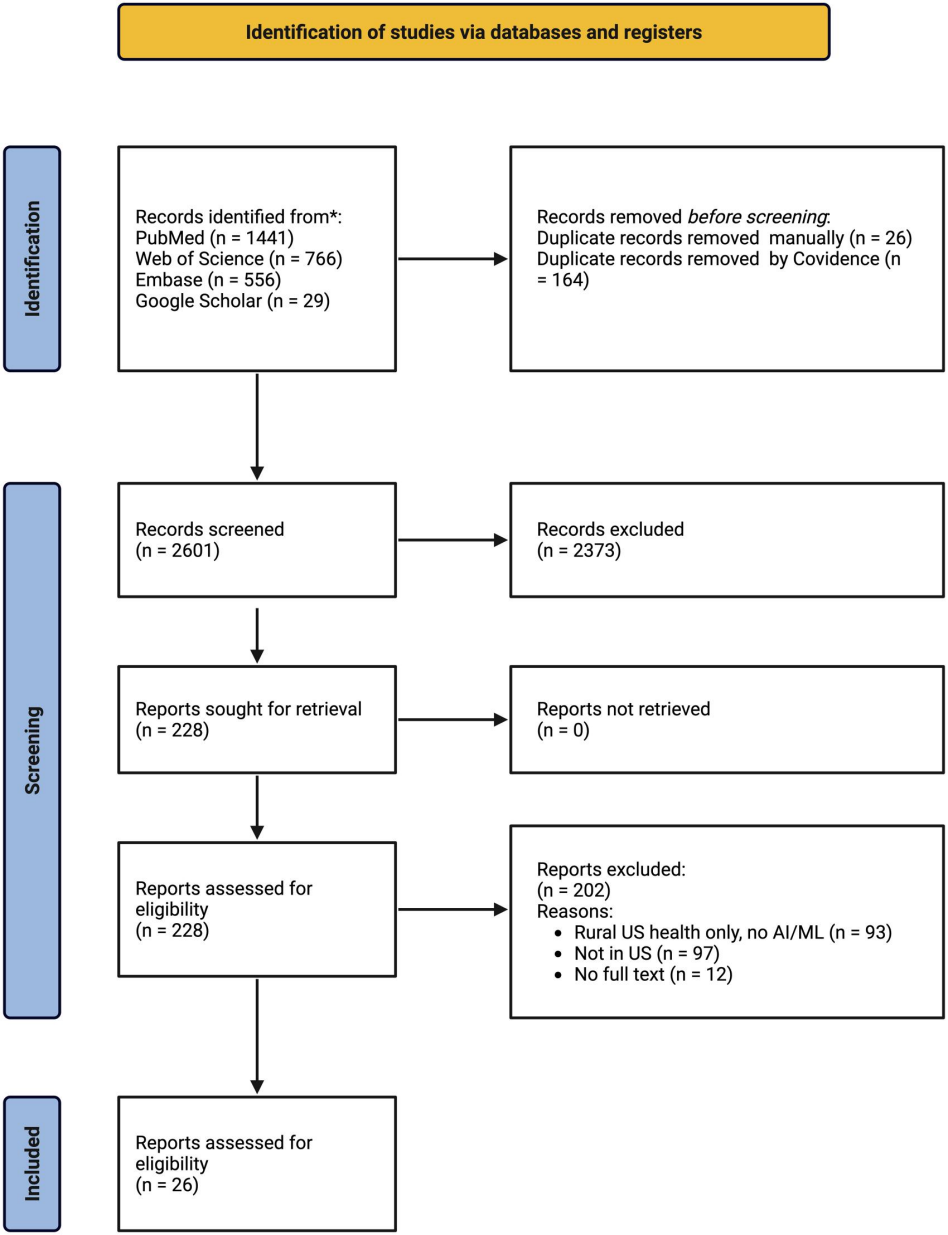
PRISMA scoping review flowchart illustrating the screening process. Created in BioRender. Brown, K. (2025) https://BioRender.com/jt6i6tu.

**Figure 2.**
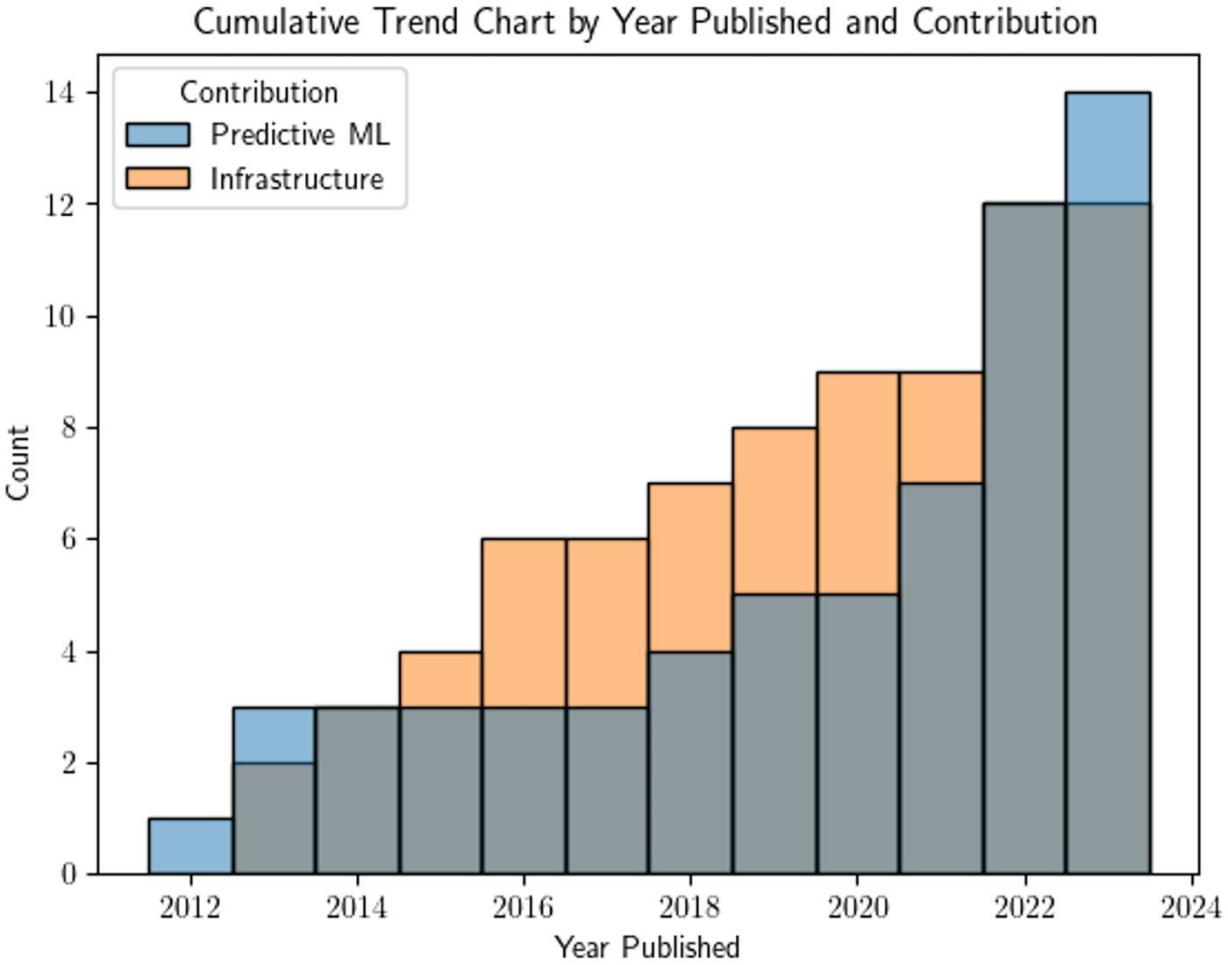
Cumulative trend chart comparing predictive ML and infrastructure contributions by year.

Our search strategy returned 2792 studies. After removing duplicates, 2601 studies were included in our initial title and abstract screening. This first screening step eliminated 2373 studies, leaving 228 for full text review and 26 studies meeting inclusion criteria after full text screening (Figure 1). Many studies (*n* = 22, 85%) were journal articles and the remaining 15% (*n* = 4) were papers published in conference proceedings. Table 2 provides an overview of the 14 papers that discussed predictive AI models and Table 3 describes the 12 papers that discussed data or research infrastructure. No studies discussed generative AI models trained, evaluated, or deployed in a rural setting. Figure 2 shows how the literature evolved over time. Between 2010 and 2013, research focused on predictive AI over data or research infrastructure. By 2015, there was an equal number of predictive modeling and infrastructure papers. Work in data and research infrastructure dominated this landscape between 2015 and 2021. During this time, total work in predictive AI for rural US health was initially stagnant (2014-2017) before seeing slight growth (2018-2021). In 2022, both predictive AI and infrastructure literature increased substantially, and from 2022, predictive AI studies again outnumbered infrastructure contributions.

**Table 2.** Summary chart describing the data collection for the predictive ML papers included in our review.

	Year	Type	Data	ML type	Validation type	Metrics	Medical specialty	Prediction task	Lifecycle stage(s)	Formal definition of rurality used	Information provided to estimate rurality
^47^	2013	CP	EHR, Other	Tree Ensemble	Holdout (bootstrapped)	C-statistic	Cardiology	30-day readmission for heart failure patients	Design, Develop	None	State region
^48^	2022	JA	EHR	Tree Ensemble	Holdout (1×)	Root mean squared error, Pearson’s correlation	Orthopedic Surgery/LOS Prediction	LOS prediction after total joint replacement including hip, knee, and shoulder	Design, Develop	Population Density	N/A
^49^	2021	JA	Other	Deep Learning (LSTM)	External Validation (Temporal)	Discounted cumulative grain (DCG)	COVID-19	Recommend counties of increased incidences for intervention	Design, Develop, Deploy	RUCC	N/A
^50^	2019	JA	PDB	Unsupervised Learning	None performed	N/A	Operations	Calculating efficiency of the provision of a single health service	Design	Population Density, US Census designation, FQHC status	N/A
^51^	2012	JA	EHR	Logistic regression	External Validation (Geographic)	TPR/Sensitivity/Recall, Specificity, C-statistic/AUROC, FPR, FNR, PPV, NPV	Trauma	massive blood transfusion protocol for trauma patients	Design, Develop	Population Density	N/A
^52^	2021	JA	EHR	Tree Ensemble	Holdout (1x)	Accuracy, F1-Score, Precision, TPR/Sensitivity/Recall, Specificity, C-statistic	COVID-19	Predict extent of healthcare utilization by COVID positive patients	Design, Deploy	RUCA	N/A
^72^	2023	JA	EHR	Logistic regression, Linear Regression	Clinical trial	Correlation between predicted/true events	Cardiology	Cardiovascular disease risk management	Deploy	None	Hospital System
^53^	2013	JA	EHR	Logistic regression	External Validation (Temporal)	TPR/Sensitivity/Recall, Specificity, PPV, NPV	Internal Medicine	Predict risk of 30 day readmission	Design, Develop	Region Population	N/A
^54^	2022	JA	PDB	Tree Ensemble, Single DT	None performed (Variable Importance)	No quantitative ML metrics	Oncology	Predicting breast cancer tumor stage at diagnosis by county	Design, Develop	RUCC, Region Population, Urban Influence Code	N/A
^55^	2018	CP	EHR, Other	Logistic regression, Single DT	k-fold cross-validation	C-statistic	Obstetrics	Prediction of preterm birth	Design, Develop	None	None
^56^	2022	JA	EHR	Logistic regression	Clinical trial	No quantitative ML metrics	Endocrinology	Pre-diabetes detection	Develop, Deploy	RUCA	N/A
^57^	2022	JA	EHR, Clinical Text	Tree Ensemble, Non-tree ensemble, Logistic regression, Single DT	External Validation (Temporal), k-fold cross-validation	Accuracy, F1-Score, Recall, Precision, G-score, Balanced Accuracy	Primary Care	Identify medical reason for patient appointment	Design, Develop	FQHC status	N/A
^58^	2022	JA	EHR, Clinical Text	Tree Ensemble, Gaussian Process, KNN, Unsupervised Learning	k-fold cross-validation	Accuracy, F1-Score, Recall, Precision, G-score, Balanced Accuracy	COVID-19	Predicting covid-19 test results based on testing reason	Design, Develop	FQHC status	N/A
^59^	2023	JA	EHR	Tree Ensemble, Linear Regression	Holdout (1x)	C-statistic	Neurology	Predicting age of “young strokes”	Develop	None	State region, Hospital System Capabilities

Abbreviations: CP = Conference Proceedings; JA = Journal Article; PDB = publicly available, non-EHR database. Data denoted as other contains billing data, health department data or a combination of biological and environmental data.

**Table 3.** Summary chart describing the data collection for the infrastructure papers included in our review.

Citation	Year	Article type	Data	Medical specialty	Formal definition of rurality used	Information provided to estimate rurality
^60^	2022	JA	PDB, surveys, reporting, administrative data, non-traditional health measures	N/A	None	County
^61^	2019	JA	PDB	N/A	None	State region County
^62^	2020	JA	N/A	N/A	None	State region
^63^	2014	JA	EHR, Clinical Text	N/A	FQHC status	N/A
^64^	2013	JA	EHR, Clinical Text	N/A	None	State region
^65^	2016	CP	EHR	Endocrinology	None	State region
^66^	2015	CP	EHR, Clinical Text, Operational, Financial, Quality Measures	N/A	None	None
^67^	2018	JA	EHR, Clinical Text	Cardiology	None	County
^68^	2013	JA	EHR	N/A	None	State region
^69^	2022	JA	EHR, Clinical Text, PDB, billing data	COVID-19	None	State region
^70^	2022	JA	EHR, PDB	Oncology	HRSA Eligibility, Region Population	N/A
^71^	2016	JA	N/A	Quality Improvement	FQHC status	N/A

CP = Conference Proceedings; JA, Journal Article; PDB = Public, non-EHR database.

We also examined the medical specialty for both predictive modeling and infrastructure papers. For studies of predictive AI, models were commonly related to the COVID-19 pandemic (*n* = 3, ∼21% of predictive AI subset, 4% total) and cardiology outcomes (*n* = 2, ∼14% of predictive AI subset, 8% total). The remaining 9 (64% of predictive AI subset, 35% total) explored a wide variety of medical specialties, including endocrinology, internal medicine, neurology, obstetrics, oncology, primary care, trauma, orthopedic surgery, and hospital operations. Half of the infrastructure papers (*n* = 6, 23% total) were not concerned with a single specialty but focused on assimilating EHR data from inpatient and outpatient settings. The remaining infrastructure papers included tools focused on supporting analyses for COVID-19, cardiology, endocrinology, oncology, public health, and quality improvement.

**Figure 3.**
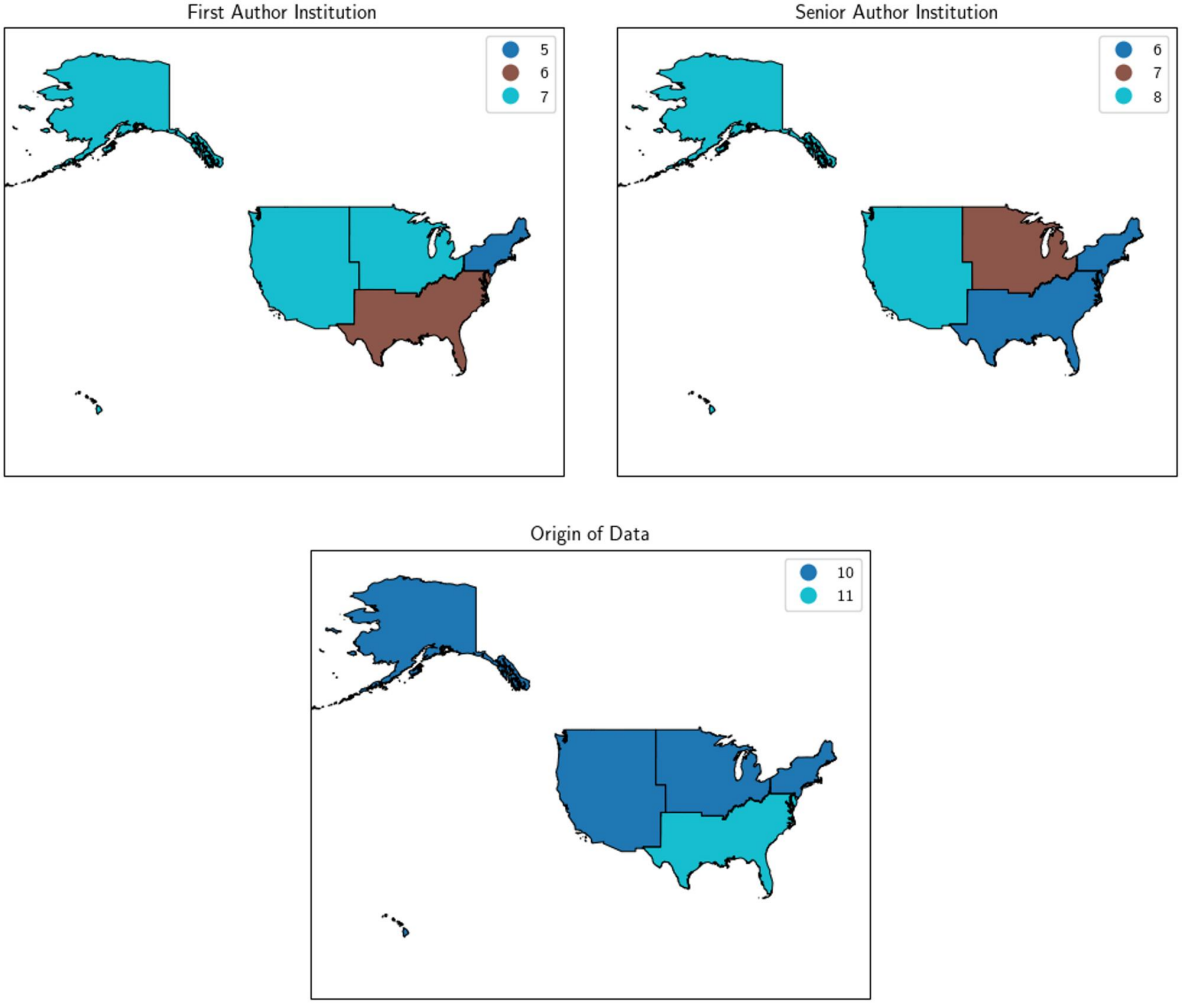
Geographic heatmaps of first author, senior author, and data origin per paper.

Studies of AI in rural health included researchers and communities from across the US (see Figure 3), highlighting broad interested in AI across diverse rural communities. Approximately 8% (*n* = 2) of studies did not list the state or US region in which data were collected and one study included data from Puerto Rico—a US territory. Of the papers that addressed the development or validation of predictive AI models, 79% (*n* = 11, 42% total) were authors by researchers from the same state from which some or all of the data originated.

### Predictive AI models

We collected the type of predictive algorithms commonly used in AI research in the rural US. This information could help to elucidate the computational capabilities of rural healthcare centers and the diffusion of advanced modeling methods. The most common predictive algorithms were tree ensembles such as gradient-boosted trees or random forests (*n* = 7/14, 50% of predictive AI subset). Logistic regression (*n* = 6/14, 43%) and non-ensemble decision trees (*n* = 3/14, 21%) were the next most frequent, followed by linear regression (*n* = 2/14, 14%), and various forms of unsupervised learning (*n* = 2/14, 14%). Adaboost (a non-tree-based ensemble), deep learning in the form of long short-term memory (LSTM) networks, Gaussian processes, and k-nearest neighbors were each used in a single study. Three-fourths of studies (79%, *n* = 11) conducted model validation, one study pursued multiple forms of model validation, and 2 studies did not present validations. The most common validation procedures (*n* = 3/14 papers each, 21%) were temporal data splitting, random data splitting with a single holdout test set, and k-fold cross-validation. Two papers (14%) evaluated a predictive model as part of a clinical trial. Finally, one paper (7%) performed geographic external validation. The most common validation metric was binary area under the receiver operating characteristic curve (AUROC, equivalent *C*-statistic).

The types of data included in developed or validated models can also elucidate the capabilities of rural healthcare centers to apply AI advances to benefit their local populations. Of the papers discussing predictive models, almost 80% (*n* = 11/14) used structured EHR data. The next most common data source was clinical narrative text (eg, notes and clinical summaries) and publicly available, non-EHR data sources, each being used in 2 papers (14%). One paper each (7%) used billing data, health department data or a combination of biological and environmental data. In 71% of predictive modeling papers (*n* = 10/14), data originated from a resource combining information from multiple rural medical providers, and the remaining 29% (*n* = 4/14) used data from a single rural medical center. Four papers (29%) used data originating from an independent, non-academic medical center, and 14% (*n* = 2/14) used data from an academic medical center with rural satellite sites or data not associated with a medical center (ie, external data sources/publicly available datasets).

Of the papers discussing predictive models, 10 (72%) applied a definition of rurality to contextualize their study. The most common definitions used were whether the site was a federally qualified health center (FQHC) and population density. Population density is clear measure of rurality. However, facilities designated as FQHC may be in rural or urban areas, with FQHC alone only indicating community disadvantage rather than community size. For those studies self-identifying as occurring in a rural setting but only providing FQHC as a defining characteristic, we confirmed rurality of the study location using Census data to confirm whether population density of the described area was less than 500 people per square mile. Studies also documented rurality with governmental classifications systems such as the RUCC and rural-urban commuting area (RUCA) codes.

Two studies^49^^,^^52^ specifically included both urban and rural data to compare model performance across this dimension. Differences in model performance between rural and urban subpopulations were not always noted; however, when noted and statistically significant, models performed better in urban populations than in rural populations. Two studies^48^^,^^51^ compared the performance of novel or existing models in rural areas to previously reported model performance information—significant differences in model performance between rural and urban subpopulations were noted, with both studies advocating for region- and population-specific model development strategies.

To understand the maturity of AI implementations in rural healthcare, we investigated the phases of the AI development lifecycle associated with each research study. Of the predictive AI papers, 79% (*n* = 11) contained an aspect of the design phase, 79% (*n* = 11) contained an aspect of the develop phase, and 29% (*n* = 4) contained an aspect of the deploy phase. Two papers (14%) could be considered as implementation studies. Both implementation studies^56^^,^^72^ noted difficulties with adoption of the AI tools due to limited financial and technical resources.

### Infrastructure

Our review highlighted several initiatives, such as the Appalachian Informatics Platform,^62^ that could support AI development and dissemination in rural healthcare settings. Most such studies were undertaken in the western US (*n* = 7/12, ∼58%). The majority of infrastructure papers (11 of 12, 92%) accessed structured EHR data as the primary data source. Two papers included publicly available data or clinical narrative text (17% each), and one paper (8%) accessed data from a health information exchange, including public health departments laboratory data, billing, operational, and quality measures in their database. All papers used data combined across multiple sources.

All infrastructure papers focused on data collection. Data harmonization (*n* = 9/12, 75%) was a common concern and half of the infrastructure papers (*n* = 6/12) sought to provide data visualization tools. One paper (8%) aimed to enable data sharing across institutions. Most papers (*n *= 9/12, 75%) did not explicitly mention support for AI as a current or future use case of the infrastructure. Two infrastructure papers (*n* = 2/12) included tools to support predictive analytics and one paper demonstrated the development of an AI model. Two papers explicitly noted AI modeling as a future direction for the described or evaluated infrastructure tools. Of papers discussing infrastructure development, 3 (25%) reported a standard or non-standard definition of rurality, with FQHC used most frequently. The remaining 9 papers disclosed the availability geographic information that could be used to estimate a standard definition of rurality.

### Challenges to AI in rural healthcare

Table 4 presents challenges for AI development and deployment in rural settings that are explicitly mentioned in the “Discussion” and “Limitations” sections of each of the reviewed studies. The most common acknowledged challenges were a lack of reliable, high quality data sources and small data volumes. Small data volumes represent an important challenge to robust model development, rigorous model validation, and successful localization of AI models developed in other communities. The next most highlighted challenge was a lack of data science expertise at rural healthcare facilities. The existing urban-rural healthcare divide, lack of data harmonization and low data quality, and lack of technical infrastructure were also noted. Other challenges included differences in disease prevalence and patient demographics across geographic regions, difficulties in organizational/community engagement, lack of medical staff or medical staff training in informatics tools, and the need for AI governance. These challenges highlight technical, organizational, and workforce related barriers to AI research and adoption that may require more robust guidance and recommendations for AI-enabled healthcare; geographic diffusion of expertise through workforce development programs; and broad investments in infrastructure and data sharing capacity.

**Table 4.** Distribution of challenges present in each paper covered in our scoping review.

Challenge	Number of papers (%)
Lack of reliable data sources/volume	13 (50%)
Lack of data science expertise	8 (30.77%)
Existing urban-rural healthcare divide	7 (26.92%)
Lack of data harmonization	7 (26.92%)
Lack of technical infrastructure	7 (26.92%)
Differences in disease prevalence	5 (19.23%)
Differences in demographics	4 (15.38%)
Difficulties in organizational/community engagement	4 (15.38%)
Lack of work evaluating clinical prediction models to rural communities	3 (11.54%)
Lack of staff or staff training	2 (7.69%)
Need for AI governance	2 (7.69%)
Lack of non-data science, non-clinical expertise	1 (3.85%)

## Discussion

We now synthesize our results in the context our research questions. First, we discuss how research in AI has evolved. Then, we discuss trends in tasks and techniques, gaps in the research, and challenges noted in the current literature. Finally, we present limitations of this scoping review.

### Evolution of research

Research on AI solutions for rural healthcare mirrors the adoption of EHR systems in the rural US. After an initial surge in research in 2013 and 2014, new contributions in rural AI development slowed until 2022 and then increased, in part, as a response to the coronavirus pandemic. This initial interest in AI development correlates with the HITECH Act^41^ incentivizing the adoption and use of EHR systems in American healthcare systems—including in the rural US—and the pandemic reigniting investments in AI and EHR extensions such as telehealth tools. Rural healthcare centers, however, continue to lag behind urban sites in EHR^73^ and telehealth adoption.^74^ Our review highlights a continued need for investments in EHR data infrastructure, workforce expertise, and computational resources are needed to advance AI in rural healthcare. As research into rural healthcare informatics and AI advances, the field’s broader understanding of adoption and impact on care would also benefit from consistent use of standardized definitions of rurality.^75^

### Tasks and techniques

For predictive AI models, applications in rural healthcare most commonly targeted resource allocation and distribution. We noted several attempts to predict resource utilization surrounding COVID-19 testing needs and case distributions. This was likely pursued as an attempt to predict where public health agencies could efficiently direct resources to help mitigate strain induced by surging caseloads and limited healthcare capacity. There were few AI solutions targeting acute medical events faced by rural patients, such as trauma and stroke. Outcomes are worse for rural patients suffering from these events^7^^,^^8^ and as such these conditions pose an opportunity for AI to improve care for rural patients. The limited availability of clinicians trained in these time-critical specialties in rural areas often necessitates patients with such conditions be transferred to larger, more resourced hospitals. We posit that it is possible to improve health outcomes for these patients by developing and evaluating targeted AI models for these scenarios to improve accessibility of care resources, speed diagnoses, and support effective care transfers.

Practical limitations may be influencing and limiting the types of AI models evaluated in rural US medical facilities. The most frequent models employed, tree-based ensembles such as random forests and gradient-boosting trees, are common and powerful algorithms that do not require the same levels of energy or computational overhead as neural networks and LLMs.^76^ This means such models may be more feasible for implementation at rural medical centers where computational resources may be limited. We observed few explorations of deep learning and advanced neural network models, including generative AI such as LLMs, in rural health care settings. Deep learning was utilized in one paper to predict counties in West Virginia likely to face an increase in COVID-19 infections using non-text epidemiological data.^49^ No generative AI models were captured in our review. We also did not find studies translating advances in AI-based pathology and radiology diagnostic tools to rural communities. These well-researched and successful use cases of deep learning^77–80^ could be particularly useful at rural medical centers with limited specialty providers and support more efficient remote care. Rural healthcare centers may outsource pathology and radiology services^81^; however, we did not see any evaluation studies of such implementations. Specialized deep learning models, however, require intensive and expensive computational power, which may be infeasible for small, rural medical centers. We note this lack of research into deep learning for rural US healthcare has introduced a rural-urban divide in AI technologies—widening the existing rural-urban healthcare divide. Unfortunately, this divide is likely to expand if research into generative AI does not include evaluating performance for rural US healthcare and improving accessibility to underserved communities.

### Research gaps

Our review highlighted few studies of AI moving beyond the design and develop stages, leaving a clear gap in our understanding of how to deploy and sustain AI models in rural settings. Several challenges noted in the reviewed studies may provide insight into this lack of translation from research to implementation. Multiple papers noted a lack of technical infrastructure and dedicated staff to train and validate AI solutions. This may be attributed to a lack of funding, which was another common barrier to adoption. Limited data resources and sample sizes for training complex models, evaluating model performance, and measuring the impact of deployed tools also pose a unique challenge for rural healthcare facilities. Further research is needed to enhance the translation of state-of-the-art modeling techniques into effective AI tools for use in the rural US, including exploring partnerships between academic medical centers and rural communities; solutions to logistic challenges of such partnerships, including data and resource sharing; and exploration of novel data augmentation techniques to enhance data volumes.

Validation of AI tools in the rural US was underwhelming. The most common form of model validation was a single random holdout test set. In this paradigm, most available data is used for model training while a sample subset of unseen data is reserved for evaluation. This approach can provide overly optimistic indications of model performance and obscure indications of model overfitting if the test set by random chance provides an advantageous or disadvantageous split between training and validation data.^82^ This may be of particular concern for small datasets from rural facilities where test sets are small and subject to high variability. Only one paper used multiple holdouts via bootstrapping, and 3 papers use a form of k-fold cross-validation. These techniques train and evaluate models multiple times using random partitions of the data to help provide more realistic assessments of model performance; however, these approaches may not address geographic or temporal generalizability. External validation is always recommended and was considered in 4 papers that performed multi-site external evaluation or temporal splitting for validation.

### Challenges to AI research

Reviewed studies highlighted a lack of reliable data sources or limited data volume as a potential challenge in developing and adopting AI. Patient-level EHR data was often limited to specific medical centers, which can only provide small sample sizes in rural communities. While existing multisite patient-level EHR databases such as All of Us^46^ or electronic ICU^83^ contain proxies for rurality such as site ZIP-3 codes or site size, these sources are not widely used for research in AI for the rural US. Moreover, these databases may not reflect demographic or medical event prevalence of a specific rural area, a widely noted concern with model development and evaluation.^84^ Synthetic data generation^85^ and federated learning^86^ are 2 technical approaches that could help mitigate these sample size and data representativeness concerns, but such approaches have yet to be applied to support AI in rural health and may require additional computational and analytic staff support.

### Limitations

We did not perform a complete critical appraisal of individual sources of evidence. This is a burgeoning subfield of informatics and the literature does not yet warrant a critical appraisal of each study’s methods. The goal of this scoping review is to summarize of the current state of the literature. Future work in a systematic review when the literature has matured should consider a more complete critical appraisal of the literature. Additionally, we acknowledge that the limited sample size of retrieved articles limits the generalizability of some of our findings. This will also require more maturity in the available body of research. There is a need to further quantify the impact of cost, data scarcity, and other challenges on the performance and adoption of AI in rural healthcare and evaluate strategies to help mitigate these concerns.

## Conclusion

Rural medical centers are overburdened and understaffed, making the promise of efficiency and improved care quality through AI tools particularly critical. In this work, we performed a scoping review of healthcare AI tools and infrastructure in the rural United States. Most predictive AI research focused on models for resource allocation using structured EHR data and common ML algorithms, such as ensembles of tree-based models, decision trees, and regression. The rural US faces challenges in data volume and quality, leading to less robust evaluation of predictive models. The lack of technical infrastructure, data science staffing, and funding have led to a growing urban-rural divide in AI research. Narrowing this divide is a growing necessity, especially with the rise of generative AI and the risk of further exacerbating the urban-rural divide if effective generative AI tools are not broadly available to all communities. Given the limited research into rural use of clinical AI and the challenges to deploying AI in rural settings, research and operational institutional partnerships, as well as policy initiatives, will be necessary to realize the promise of healthcare AI for all individuals and communities across the United States. Next steps for rural AI research include tailored methods for clinical AI model localization, validation, and monitoring; recommendations for AI implementation in resource limited settings; frameworks for multi-site coordination and AI governance; and evaluations of generative AI solutions in rural settings, including needs assessments, workflow integration, and impact assessments.

## Supplementary Material

ocaf206_Supplementary_Data

## Data Availability

Minimum necessary data to generate results presented in this paper are contained in this manuscript and Supplementary Materials. An Open Science Foundation repository is available at the following link: https://osf.io/rz2xe/?view_only=66f708fb7c6b4cfda582c7d1e2e5378d
