# Supplementary material for: Gaps in artificial intelligence research for rural health in the United States: a scoping review
Source: J Am Med Inform Assoc. 2025 Nov 24;33(2):509–20. doi: 10.1093/jamia/ocaf206 (PMC12844595; doi:10.1093/jamia/ocaf206)
Supplement: ocaf206_Supplementary_Data [file ocaf206_supplementary_data.docx]

**Gaps in Artificial Intelligence Research for Rural Health in the United States: A Scoping Review**

Katherine E. Brown, PhD and Sharon E. Davis, PhD

Department of Biomedical Informatics, Vanderbilt University Medical Center, Nashville, TN

**Corresponding Author:**

Katherine E. Brown, PhD

2525 West End Ave, Suite 1475

Nashville, TN 37203

katherine.brown@vumc.org

**S1. Preferred Reporting Items for Systematic reviews and Meta-Analyses extension for Scoping Reviews (PRISMA-ScR) Checklist**

Table S1. PRISMA-ScR checklist for our literature review.

| **SECTION** | **ITEM** | **PRISMA-ScR CHECKLIST ITEM** | **REPORTED ON PAGE #** |
| --- | --- | --- | --- |
| **TITLE** | | | |
| Title | 1 | Identify the report as a scoping review. | 1 |
| **ABSTRACT** | | | |
| Structured summary | 2 | Provide a structured summary that includes (as applicable): background, objectives, eligibility criteria, sources of evidence, charting methods, results, and conclusions that relate to the review questions and objectives. | 2 |
| **INTRODUCTION** | | | |
| Rationale | 3 | Describe the rationale for the review in the context of what is already known. Explain why the review questions/objectives lend themselves to a scoping review approach. | 2-3 |
| Objectives | 4 | Provide an explicit statement of the questions and objectives being addressed with reference to their key elements (e.g., population or participants, concepts, and context) or other relevant key elements used to conceptualize the review questions and/or objectives. | 3 |
| **METHODS** | | | |
| Protocol and registration | 5 | Indicate whether a review protocol exists; state if and where it can be accessed (e.g., a Web address); and if available, provide registration information, including the registration number. | 5 |
| Eligibility criteria | 6 | Specify characteristics of the sources of evidence used as eligibility criteria (e.g., years considered, language, and publication status), and provide a rationale. | 5-6 |
| Information sources* | 7 | Describe all information sources in the search (e.g., databases with dates of coverage and contact with authors to identify additional sources), as well as the date the most recent search was executed. | 5-6 |
| Search | 8 | Present the full electronic search strategy for at least 1 database, including any limits used, such that it could be repeated. | 5, S1 |
| Selection of sources of evidence† | 9 | State the process for selecting sources of evidence (i.e., screening and eligibility) included in the scoping review. | 5-6 |
| Data charting process‡ | 10 | Describe the methods of charting data from the included sources of evidence (e.g., calibrated forms or forms that have been tested by the team before their use, and whether data charting was done independently or in duplicate) and any processes for obtaining and confirming data from investigators. | 6 |
| Data items | 11 | List and define all variables for which data were sought and any assumptions and simplifications made. | 7 |
| Critical appraisal of individual sources of evidence§ | 12 | If done, provide a rationale for conducting a critical appraisal of included sources of evidence; describe the methods used and how this information was used in any data synthesis (if appropriate). | N/A |
| Synthesis of results | 13 | Describe the methods of handling and summarizing the data that were charted. | 6 |
| **RESULTS** | | | |
| Selection of sources of evidence | 14 | Give numbers of sources of evidence screened, assessed for eligibility, and included in the review, with reasons for exclusions at each stage, ideally using a flow diagram. | 7, Figure 1 |
| Characteristics of sources of evidence | 15 | For each source of evidence, present characteristics for which data were charted and provide the citations. | 7-9, Figures 2-3, Tables 2-4 |
| Critical appraisal within sources of evidence | 16 | If done, present data on critical appraisal of included sources of evidence (see item 12). | N/A |
| Results of individual sources of evidence | 17 | For each included source of evidence, present the relevant data that were charted that relate to the review questions and objectives. | 7-9, Tables 2-3 |
| Synthesis of results | 18 | Summarize and/or present the charting results as they relate to the review questions and objectives. | 10-12 |
| **DISCUSSION** | | | |
| Summary of evidence | 19 | Summarize the main results (including an overview of concepts, themes, and types of evidence available), link to the review questions and objectives, and consider the relevance to key groups. | 10-12 |
| Limitations | 20 | Discuss the limitations of the scoping review process. | 13 |
| Conclusions | 21 | Provide a general interpretation of the results with respect to the review questions and objectives, as well as potential implications and/or next steps. | 13 |
| **FUNDING** | | | |
| Funding | 22 | Describe sources of funding for the included sources of evidence, as well as sources of funding for the scoping review. Describe the role of the funders of the scoping review. | 16 |

**S2. Search Terms**

Table S2. Search terms used to query PubMed, Embase, and WebOfScience and number of results retrieved from each database.

| Database | Search Term | # Results (before removing duplicates) |
| --- | --- | --- |
| PubMed | ("rural health"[MeSH Terms] OR "Rural Health Services"[MeSH Terms] OR "community medicine"[MeSH Terms] OR "rural population"[MeSH Terms] OR "community health centers"[MeSH Terms]) AND ("artificial intelligence"[MeSH Terms] OR "data science"[Text Word] OR "clinical decision support"[Text Word] OR "informatics"[MeSH Terms]) AND ("humans"[MeSH Terms]) AND (2010:2024[pdat]) | 1441 |
| Embase | ('rural health'/exp OR 'rural health care'/exp OR 'community medicine'/exp OR 'health center'/exp OR 'rural population'/exp) AND ('artificial intelligence'/exp OR 'data science'/exp OR 'clinical decision support system'/exp OR 'information science'/exp) AND 'human'/exp | 556 |
| WebOfScience | (TS="rural health" OR TS="Rural Health Services" OR TS="community medicine" OR TS='rural population' OR TS='community health centers') AND (WC="Computer Science, Artificial Intelligence" OR TS="data science" OR TS="clinical decision support" OR TS="informatics") | 766 |

Table S3. Population-concept-context chart for retrieved studies

| Population | Rural medical center is defined as any organization (in-patient or out-patient) providing patient care in a predominantly rural area |
| --- | --- |
| Concept | Included literature must discuss:   - AI development or use - Infrastructure to facilitate AI research |
| Context | We define rurality as:   - Population density < 500 people per square mile - Rural Urban Continuum Code > 3 - Rural or Borderline Rural classification in CMS Ambulance Fee Schedule |
